# Patients with juvenile idiopathic arthritis have decreased clonal diversity in the CD8^+^ T cell repertoire response to influenza vaccination

**DOI:** 10.3389/fimmu.2024.1306490

**Published:** 2024-05-30

**Authors:** Sara E. Sabbagh, Dipica Haribhai, Jill A. Gershan, James Verbsky, James Nocton, Maryam Yassai, Elena N. Naumova, Erin Hammelev, Mahua Dasgupta, Ke Yan, Jack Gorski, Calvin B. Williams

**Affiliations:** ^1^ Division of Rheumatology, Department of Pediatrics, Medical College of Wisconsin, Milwaukee, WI, United States; ^2^ Divison of Hematology/Oncology, Department of Pediatrics, Medical College of Wisconsin, Milwaukee, WI, United States; ^3^ Versiti Wisconsin, Blood Research Institute, Milwaukee, WI, United States; ^4^ Division of the Nutrition Epidemiology and Data Science, Friedman School of Nutrition Science and Policy, Tufts University, Boston, MA, United States; ^5^ Division of Quantitative Health Sciences, Department of Pediatrics, Medical College of Wisconsin, Milwaukee, WI, United States

**Keywords:** CD8 + T cells, T cell repertoire, influenza vaccination, clonotypes, juvenile idiopathic arthritis, clonotype diversity

## Abstract

Recurrent exposures to a pathogenic antigen remodel the CD8^+^ T cell compartment and generate a functional memory repertoire that is polyclonal and complex. At the clonotype level, the response to the conserved influenza antigen, M1_58–66_ has been well characterized in healthy individuals, but not in patients receiving immunosuppressive therapy or with aberrant immunity, such as those with juvenile idiopathic arthritis (JIA). Here we show that patients with JIA have a reduced number of M1_58–66_ specific RS/RA clonotypes, indicating decreased clonal richness and, as a result, have lower repertoire diversity. By using a rank-frequency approach to analyze the distribution of the repertoire, we found several characteristics of the JIA T cell repertoire to be akin to repertoires seen in healthy adults, including an amplified RS/RA-specific antigen response, representing greater clonal unevenness. Unlike mature repertoires, however, there is more fluctuation in clonotype distribution, less clonotype stability, and more variable IFNy response of the M1_58–66_ specific RS/RA clonotypes in JIA. This indicates that functional clonal expansion is altered in patients with JIA on immunosuppressive therapies. We propose that the response to the influenza M1_58–66_ epitope described here is a general phenomenon for JIA patients receiving immunosuppressive therapy, and that the changes in clonal richness and unevenness indicate a retarded and uneven generation of a mature immune response.

## Introduction

Juvenile idiopathic arthritis (JIA) is the most common chronic inflammatory rheumatic disease in childhood. It is characterized by chronic joint inflammation due to immune dysregulation and is treated with immunosuppressive medication. Although aggressive treatment of JIA can improve long-term outcomes (1), it may increase the risk of infection (2). Due to iatrogenic and inherent immunocompromise, much emphasis has been placed on vaccination of patients with chronic inflammatory rheumatic diseases (3, 4). However, data is limited and mixed regarding vaccine efficacy in this patient population (5–12). This has come to the forefront given the outbreak of the SARS-CoV-2 pandemic and development of SARS-CoV-2 vaccines. There is accumulating evidence that immunocompromised patients may not mount an adequate antibody response to SARS-CoV-2 vaccines (13, 14) and some clinicians have anecdotally held therapeutic immunosuppression to optimize SARS-CoV-2 vaccine efficacy (15). Overall, the immunogenicity of vaccines given to children with iatrogenic and/or inherent immunosuppression is poorly described, making clinical recommendations challenging.

The development of an adequate antiviral immune response following antigen exposure depends on clonal expansion of pathogen-specific memory T cells (16). The clones that expand are defined by their unique T cell receptor (TCR) alpha and beta chain third complementarity-determining regions (CDR3) nucleotide sequences and are referred to as clonotypes. T cell clonotype diversity is generated during the recombination events which affect the final nucleotide sequences in the TCR alpha and beta chain CDR3. Following repeated encounters with a foreign antigen, the T cell memory compartment becomes remodeled, generating a functional memory repertoire that is polyclonal and complex. Although our understanding of the SARS-CoV-2-specific T-cell response remains in its infancy (16), T cell clonotype response following exposure to the conserved influenza antigen, M1_58–66_ (GILGFVFTL) has been well characterized (17–29). Against the M1_58–66_ influenza antigen there is a polyclonal memory T cell recall response with primary usage of the TCR beta chain Variable19 (βV19, formerly referred to as βV17) gene (17–19) in HLA-A2 individuals. This βV19 TCR gene region has a predominant group of clonotypes with a CDR3 length of 11 amino acids (CDR3(11)) and contains an Arginine/Serine (RS), and less frequently, an Arginine/Alanine (RA) motif in the TCR (RS/RA) (17, 19, 21).

We have previously shown that in HLA-A2 healthy controls, the recall repertoire response to the conserved influenza M1_58–66_ epitope generates few RS/RA clonotypes present at high frequency, more RS/RA clonotypes at intermediate frequencies, and a large number (often over 50%) at very low frequencies (19, 27, 30). We have previously characterized T cell repertoires with clonal expansion using mathematically defined properties of *richness*, *unevenness*, and *diversity* (31). These properties within an individual TCR repertoire are broadly applicable to understanding the adaptive cell-mediated immune response in disease states.

To date, analysis of T cell clonal expansion following vaccination has not been described in patients receiving immunosuppressive therapy or with aberrant immunity, such as those with JIA. Therefore, the goal of this study was to better understand the adaptive cell-mediated immune response to vaccination in JIA patients on immunosuppressive therapies. Here we describe how the T cell recall response to the influenza M1_58–66_ epitope in HLA-A2 JIA patients receiving immunosuppressive therapy displays reduced clonal diversity compared to HLA-A2 healthy pediatric controls.

## Materials and methods

### Subjects and study design

Blood samples were obtained from neonates, pediatric healthy controls and pediatric JIA patients. Peripheral blood mononuclear cells (PBMCs) were separated by density gradient centrifugation using Ficoll Histopaque and stored at -80°C. Subjects included 5 HLA-A2 patients with polyarticular or extended oligoarticular JIA between the ages of 6 and 16, 6 healthy control children (4 females and 2 males) between the ages of 6 to 14 years and 6 HLA-A2 newborns (sex unknown) between the ages of 5 to 128 days that were undergoing corrective surgery for heart defects not associated with 22q11 deletion syndrome. Neonate samples were used as controls representative of antigen inexperienced (naïve) individuals. Healthy control children and patients with JIA were seen at the rheumatology clinic of Children’s Hospital of Wisconsin (CW) and enrolled under the protocol CW IRBnet: 116305 “Generation and decay of memory T cells in children with Juvenile Arthritis and healthy siblings following administration of trivalent inactivated influenza vaccine.” International Board Review approval was granted by CW and written informed consent was obtained. Inclusion criteria for subjects included JIA patients between the ages of 3 and 18 years old, HLA-A2 and/or HLA DR1*01 or DRB1*04, and immunosuppression longer than three weeks with any of the following medications or combinations thereof: prednisone ≥ 0.2 mg/kg daily or 10 mg daily, methotrexate ≥ 10 mg/m2/week or 10 mg/week, and/or biologic response modifier (BRM) used at standard-of-care doses for JIA. Inclusion criteria for healthy controls included any healthy child between the ages of 3 and 18 years old who are HLA-A2 and/or HLA DR1*01 or DRB1*04 positive and who do not have chronic use of systemic immunosuppressant medications. Study visits were at 0 (baseline), 1, 6, 12, 13, 18, 24, 25, 30, 36, 37, 42, 48, and 54 months with influenza vaccine administered at baseline, 12, 24, 36, and 48-month visits. Participants received the annual trivalent non-adjuvanted inactivated vaccine (Fluzone, Sanofi-Pasteur MSD, Madrid, Spain) for at least 4 consecutive years (**Supplementary Table 1**). Patients were able to enroll at any time point during the study with blood sample collection at 1 and 6-months post-vaccinations. The Physician Global Assessment (PGA) (a 10 cm visual analog scale (VAS) which rates overall disease activity with anchors of ‘0 = no activity’ and ‘10 = maximum activity’) and the Patient/Parent Global Assessment (a 0–10 VAS which ranks the child’s overall well-being) were obtained at each study visit. Medications and number of active joints (joint count) were recorded at each visit. The clinical Juvenile Arthritis Disease Activity Score (cJADAS) was computed by the sum of the PGA, the parent/child global assessment, and the active joint count in 71 joints, yielding a global score between 0 and 91.

### Human leukocyte antigen typing

HLA typing was performed by the Versiti Blood Center of Wisconsin Histocompatibility and Immunogenetics Laboratory.

### Peptide synthesis

Influenza-specific peptide, M1_58–66_ (GILGFVFTL), was synthesized in the Versiti Blood Center of Wisconsin Peptide Core.

### Expansion of influenza virus M1_58–66_-specific T cells (recall culture)

Expansion of influenza virus M1_58–66_ -specific T cells were produced as previously described (27). Briefly, thawed peripheral blood mononuclear cells (PBMCs) were cultured in R10 media (human) at 1×106 cells/ml for 24 hours. On day 2, non-adherent PBMCs were removed and cultured with recombinant IL2 (10U/ml) for 7 days. Adherent cells were cultured for 7 days with granulocyte-macrophage colony-stimulating factor (100ng/ml), IL4 (50ng/ml) and 1μM of influenza A matrix peptide M1_58–66_ (32). On day 7 of culture, peptide loaded adherent cells were irradiated (3000R) and cultured with the non-adherent PBMCs in IL2 (10U/ml) and M1_58–66_ peptide for an additional 7 days. The goal of this short 2-week culture period is to activate and expand or “recall” memory influenza matrix peptide M1_58–66_-specific T cells. These cells were then used for spectratyping and clonotyping.

### mRNA and cDNA preparation from M1_58–66_ “recalled” CD8^+^ T cells

As previously described (28), CD8^+^ T cells were isolated from PBMCs that were expanded in the M1_58–66_ recall culture using the Dynabeads™ Human CD8^+^ isolation kit (Invitrogen, Carlsbad, CA) according to the manufacturer’s direction. CD8^+^ T cell mRNA was isolated using the DynaBeads mRNA direct kit according to the manufacturer’s instruction (Invitrogen, Carlsbad, CA). cDNA was prepared using Poly T primer (^5’^ TTTTTTTTTTTTTTTTTT^3’^) and M-MLV reverse transcriptase (Invitrogen).

### Rearrangement analysis (βV19 spectratyping)

The CDR3 length analysis (spectratyping) was performed by PCR amplification of CD8^+^ T cell cDNA using the βV19 TCR primer (^5’^ CCAAAAGAACCCGACAGCTTTC^3’^) and a carboxyfluorescein (FAM)-labeled TCRβ constant (BC) region primer (^5’^CTGTGTTTGAGCCATCAGAAGC^3’^) (28, 33). All primers were synthesized by Integrated DNA Technologies (Coralville, Iowa). To obtain a representative message for βV19, different volumes of cDNA (0.25, 0.5, and 1 ul) were PCR amplified with the primer pair for 24–30 cycles. PCR products were purified using the AMPure PCR purification kit (Beckman Coulter, Brea, CA) per manufacturer’s direction. For spectratyping analysis, 1–2μl of amplified product was combined with 9μl of deionized Formamide/Liz 500 size standard (900μl Formamide + 50μl Liz standard). Samples were heat denatured at 90°C for 3 minutes and then loaded onto an ABI 3130XL Gene Analyzer (Applied Biosystems). GeneScan software (Applied Biosystems) was used for data collection. The files were analyzed using proprietary software (GeneGeek) which gives the Relative Frequency of each CDR3 length (28).

### Clonotyping

The βV19 PCR products (described above) were cloned into E. Coli using the TOPO Cloning kit (Invitrogen). Bacterial colonies were grown overnight were sent to Agencourt Bioscience (Beverly, MA) for sequencing. Each CD3 region was PCR amplified and the length 11 band was subcloned for sequencing. Clonotype sequences were analyzed using CDR3 Reader software, which counts identical sequences and assigns clonotype names according to the convention described (34).

### Measurement of IFNγ production

PBMCs were cultured as described above in the recall culture assay. After 2 weeks in culture, PBMCs were stained with anti-CD8 allophycocyanin (APC) and sorted (FACSAria cell sorter (BD Biosciences, San Jose, CA)). The sorted CD8 cells were stained with anti-CD3 fluorescein (FITC) and anti-TCR Vβ19 phycoerythrin (PE), then fixed and permeabilized for intracellular staining with anti-IFNγ. Intracellular staining was performed using the Intracellular Fixation and Permeabilization Kit per manufacturer’s direction (Invitrogen). The percentage of CD3^+^CD8^+^Vβ19^+^IFNγ^+^ cells were reported. Healthy control C1 did not have adequate sample available for this analysis.

### Repertoire measures and characteristics

For each subject, the repertoires are described by clonal richness, or the total number of unique clonotypes observed (N), and clonal unevenness. The number of times each unique clonotype occurs is also referred to as its Rank, where clonotypes with a rank of 1 are observed once, as single copies, henceforth referred to as Singletons (Ns). To gauge extremes of clonotype distribution, the highest ranking clonotype (R_max_) is used to characterize repertoire unevenness. A repertoire is thought to be robust and resilient to distortions if it is rich in species (i.e., clonotypes) and their abundance is uneven (35).

In order to correct for individual sample size, Rank, R_max,_ and Singletons are normalized for the total number of nucleotide sequences (M) or total number of unique clonotypes (N) observed in an individual, depending on which population they describe. Thus, rank is reported as Relative Rank ( $\frac{Rank}{M}$ ), R_max_ is reported as Relative R_max_ ( $\frac{Rmax}{M}$ ) and Singletons are reported as Relative Singletons ( $\frac{Ns}{N}$ ).

### Rank Frequency summary of a clonotype distribution

Repertoire distribution of the RS clonotypes can also be described using a Rank Frequency approach (19, 20, 36). In this model, the number of unique clonotypes in each Rank is reported as Rank Frequency. Rank Frequency for each group is also normalized for the total number of RS clonotypes (N) and referred to as Relative Rank Frequency ( $\frac{Rank\;Frequency}{N}$ ). The repertoire distribution can be visualized when the Relative Rank Frequency is plotted on the Y-axis and the Relative Rank is plotted on the X-axis. The data are compatible with a single component power law-like distribution and a log/log transformation of the data (log y = log a – b log x) yields a straight line, in which the parameter *a* is an intercept of a regression line and indicates the frequency of observing single-copy clonotypes, and parameter *b* is a slope of a regression line and describes the shape of the curve by indicating how rapidly the curve decays (19, 37). These measures are used to provide a general overview of the clonotype distribution between the two extremes of Singletons and R_max_.

### Diversity measures

We measure repertoire diversity drawing on definitions of species diversity in an ecosystem. Species diversity is a reflection of both species richness and species abundance, or the overall representation of a species in a system. Similarly, repertoire diversity is a reflection of clonotype richness and clonotype unevenness (R_max_), which is a reflection of clonotype abundance (31). Thus, repertoire diversity (D_c_) is an indicator of both clonal richness (N) and clonal unevenness (Relative R_max_). Repertoire diversity can be calculated as the contribution of the most frequent clonotype in the repertoire ( $\frac{Rmax}{M}$ ), as proportional to the number of clonotypes, and is estimated as $Dc=\frac{Rma\mathrm{x(N)}}{M-1}$ .

### Clonotype stability

We used a similar approach to characterize the RS/RA clonotype temporal stability within the repertoire. The stability of the clonotype is defined as the number of times it was observed across repeated measurements. Clonotypes observed at all times analyzed are considered stable and clonotypes observed only at one time unstable. Because the measurement of temporal stability is based on multiple time points, more time points should provide a better estimation of stability. To compensate for small differences in the number of time points, we introduce a Relative Stability characteristic of the repertoire in which observation at one time is equal to a stability of 0, observation at all times is equal to a Relative Stability of 1. Thus, the Relative Stability of a clonotype is calculated as: $\frac{\text{number of times observed }–\;1}{\text{maximum possible number of times observed }–\;1}.\;$ The average Relative Stability of all the clonotypes at a given rank is the average of all the individual Relative Stabilities of clonotypes at that rank. Thus, clonotypes with rank lower than six in the pooled repertoire that consists of six time points are not stable.

### Statistical analysis

Pairwise comparisons of continuous variables were made with Student’s t-test or Mann Whitney U, as appropriate. Comparisons of combined cohort data was performed using Generalized Estimating Equation with repeated measures (subjects as clusters with uniform covariate structure). Quadratic models were fitted to check the relationship between Relative Rank and Relative Rank Frequency, and to compare the two groups. All statistical analyses were performed using SAS V9.4. As this was an exploratory study, a two-sided *p* value of<0.05 was considered statistically significant.

## Results

### Patient disease characteristics and medications

Three patients had extended oligoarticular JIA and two had polyarticular disease. All patients received non-steroidal anti-inflammatory drugs (NSAIDs) and immunosuppressive therapy with methotrexate. Patient J1 and J4 also received biologic therapy (**Table 1**). Patient J4 had persistently active arthritis with a max PGA of 10, max joint count of 54, and max cJADAS score of 70 prompting addition of IV abatacept. Similarly, patient J1 also had persistently active arthritis with max PGA of 8, max joint count of 5, and max cJADAS of 17, prompting biologic therapy with multiple medications including etanercept, infliximab, and adalimumab (**Table 1**).

**Table 1:** JIA disease demographics, characteristics and medications.

Pt	Age (yrs)	Sex	Subtype	PGA_max_	P/P_max_	JC_max_	cJADAS_max_	Medications		
								DMARDs	Biologics	Other
**J1**	10	F	Ext oligo	8	8	5	17	MTX	ETN→IFX→ADA→ETN	Naproxen
**J2**	16	F	Ext oligo	1	6	1	7	MTX		Meloxicam
**J3**	15	F	Ext oligo	5	6	5	8	MTX		Naproxen
**J4**	8	F	Poly	10	6	54	70	MTX → LEF	ABT*	Naproxen, HCQ^**^
**J5**	14	M	Poly	2	2	13	17	MTX		Naproxen

“→” Medication change, * Started at 36 months of study, ** Started at 25 months of study. The maximum (worst) value of disease assessments are shown. All patients were Rheumatoid Factor negative. Pt, Patient; F, Female; M, Male; Poly, Polyarticular juvenile idiopathic arthritis; Ext oligo, Extended oligoarticular juvenile idiopathic arthritis; PGA_max_, Max physician global assessment; P/P_max_, Max patient/parent global assessment; JC_max_, Max joint count; cJDAS, clinical Juvenile Arthritis Disease Activity Score; DMARDs, Disease Modifying Antirheumatic Agents; ADA, adalimumab; IFX, infliximab; ETN, etanercept; ABT, abatacept; LEF, leflunomide; MTX, methotrexate; HCQ, hydroxychloroquine.

### Both the CDR3(11) and the RS/RA specific CDR3(11) T cell recall response have decreased clonal richness in JIA patients compared to pediatric healthy controls

**Figure 1:**
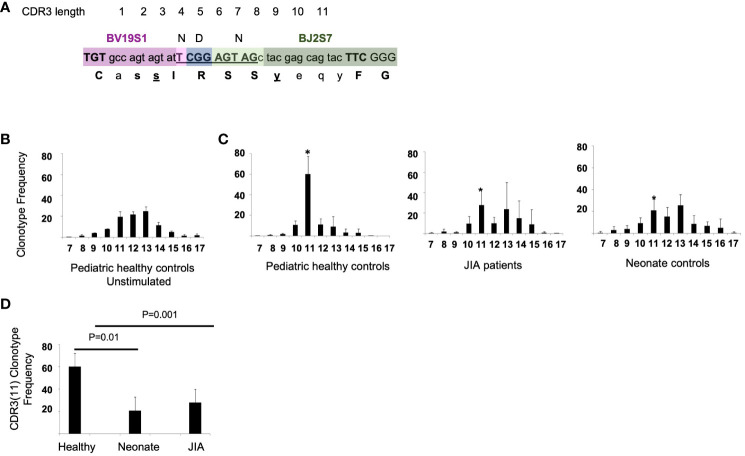
RS/RA motif and the CDR3 TCR βV19 length distribution and CDR3(11) TCR frequency in pediatric healthy controls, JIA patients, and neonates. **(A)** Drawing showing CDR3(11) and NDN amino acids “RS” in position 5 and 6 (underlined). **(B)** βV19 CDR3 length distribution from 3 healthy controls prior to stimulation with the M1_58–66_ peptide. **(C)** CDR3 length distribution of CD8^+^T cells cultured with M1_58–66_ peptide for 2 weeks from pediatric healthy controls, JIA patients and neonate controls. * Denotes the CDR3 length of 11 amino acids for each group. x-axis = CDR3 length. **(D)** Summary of the relative frequency of βV19^+^CDR3(11) RS/RA clonotypes for each cohort. Healthy controls n = 6, JIA n = 5, neonate controls n =6. Image in **(A)** redrawn from Handbook of Immunosenescence: September 2017. Naumova EN, Naumova YN and Gorski J.

There is a known adult polyclonal response of memory T cells to influenza M1_58–66_ peptide. The repertoire is reported to be highly polyclonal and complex with a few clonotypes present at high frequency, some at intermediate frequency and most (often over 50%) at very low frequencies (19, 20, 27). In response to the M1_58–66_ peptide, CD8^+^ T cells using the βV19 gene region with a CDR3(11) RS/RA motif in the NDN region are predominantly expanded (**Figure 1A**). Using this sequence as a reference, we examined the CD8^+^ T cell response to M1_58–66_ peptide in HLA-A2^+^ JIA patients and HLA-A2^+^ healthy pediatric controls.

To examine the overall βV19 clonal response independent of CDR3 length, we used spectratyping analysis, which is a quick method for determining if specific T cells are responding in a recall culture (17–19, 21, 23, 28).. For our analysis, spectratyping was done on the first sample following vaccination in pediatric healthy controls and JIA cohorts. Healthy control PBMCs that have not been stimulated in culture with M1_58–66_ peptide have a Gaussian-like CDR3 length distribution (**Figure 1B**). After a 2-week recall cultured with M1_58–66_ peptide, pediatric control patients show a focused CDR3(11) TCR βV19 repertoire. (**Figure 1C**, panel 1) The recall response of JIA patients (**Figure 1C**, panel 2) was similar to that of naïve neonate controls (**Figure 1C**, panel 3). The average CDR3 length distribution in JIA patients approximated that of unstimulated cells. We found a significant difference (p=0.01) in the clonotype frequency of CDR3(11) between healthy controls and the and JIA patients (**Figure 1D**), suggesting a reduced ability to focus the recall response in the JIA patient cohort.

**Figure 2:**
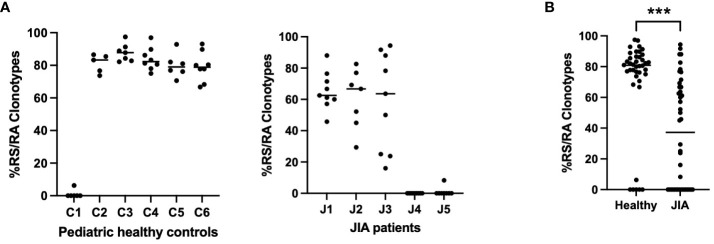
Percentage of RS/RA clonotypes within the CDR3(11) TCR repertoire in pediatric healthy controls and JIA patients. Percentage of RS/RA Clonotypes in the CDR3(11) TCR repertoire over all study time points. **(A)** Pediatric healthy controls (panel 1), JIA patients (panel 2). **(B)** Comparison of percent RS/RA clonotypes in pediatric healthy controls vs. JIA patients performed via Mann-Whitney U with two-tailed *p* value. *** = *p*< 0.001.

To further characterize the CD8^+^ T cell recall response to the M1_58–66_ peptide, we analyzed the conserved RS/RA motif in the NDN region of CDR3(11) in CD8^+^ T cells from the recalled cultures over all available time points (17–19, 21). In aggregate, we sequenced 1,566 CDR3(11) regions, of which, 866 had a conserved RS/RA motif in the NDN region. To assess the repertoire richness of each subject, we determined the frequency of CDR3(11) RS/RA clonotypes among the total number of CDR3(11) clonotypes at each visit. The response of 5 of the 6 pediatric controls was characterized by greater than 70% RS/RA clonotypes (**Figure 2A**, panel 1). Of note, one healthy control sample (C1) had only 2% RS/RA clonotypes. JIA samples had a varied percentage of RS/RA clonotypes. Three JIA patients, (J1, J2, and J3) had greater than 50% RS/RA clonotypes and two patients (J4 and J5) had less than 1.5% RS/RA clonotypes (**Figure 2A**, panel 2). The percentage of RS/RA clonotypes between all JIA patients and pediatric controls was lower (37.2 vs 80.9%, *p<* 0.001), (**Figure 2B**). Furthermore, when considering only the subset of subjects with discernable RS/RA responses (5 healthy control and 3 JIA patients), the percentage of RS/RA clonotypes in the JIA patients was lower than in the pediatric healthy controls (63.1% vs. 82.1%, *p<* 0.001). Notably, there is visible variation between study visits in the percentage of RS/RA clonotypes recalled in the JIA cohort, indicating that the richness of the repertoire recall response in JIA patients is not only reduced, but also unstable. Overall, our data show that in JIA patients the CDR3(11) recall response is reduced, and within the CDR3(11) recall response, the percentage of RS/RA clonotypes is lower. This diminution of repertoire richness suggests inability to consistently generate and focus the repertoire response in the JIA cohort.

### CD3(11) RS/RA T cell repertoires have higher Relative R_max_ and greater clonal unevenness in JIA patients compared to pediatric healthy controls

**Figure 3:**
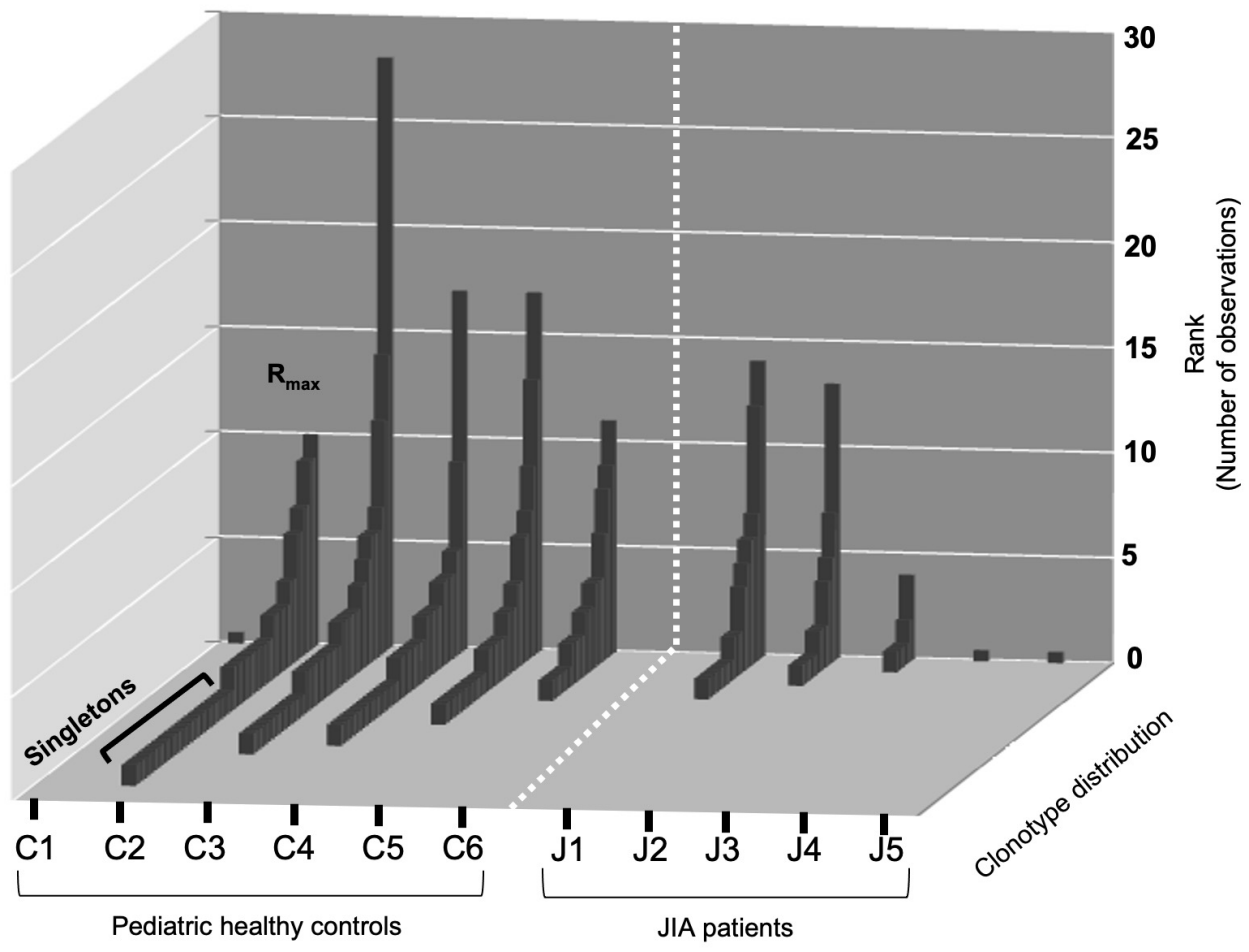
Rank distribution of RS/RA clonotypes from the TCRβV19 repertoire of CD8^+^ T cells in healthy controls and JIA patients. Distribution (R_max_ and singletons) of RS/RA encoding clonotypes from TCR βV19 repertoire of CD8^+^T cells after two weeks of culture with the M1_58–66_ peptide. The *X-axis* shows the subject ID of each cohort, the *Y-axis* shows the number of observations for each clonotype and the *Z-axis* shows clonotype distribution.

To visualize the two repertoire characteristics that frame clonotype distribution (R_max_ and Singletons), we plotted the number of observations for each clonotype (Rank) on the Y-axis and the clonotype distribution along the Z-axis for each healthy control and JIA patient in the first sample following vaccination (**Figure 3**). Healthy controls show a pattern of a one clonotype observed at a high frequency (R_max_) with many clonotypes observed once, as Singletons (Ns). The three JIA subjects with RS/RA clonotypes (J1, J2, J3) had a clonotype distribution that was similar to that of healthy controls, albeit constrained by the lower total number of RS/RA clonotypes.

**Figure 4:**
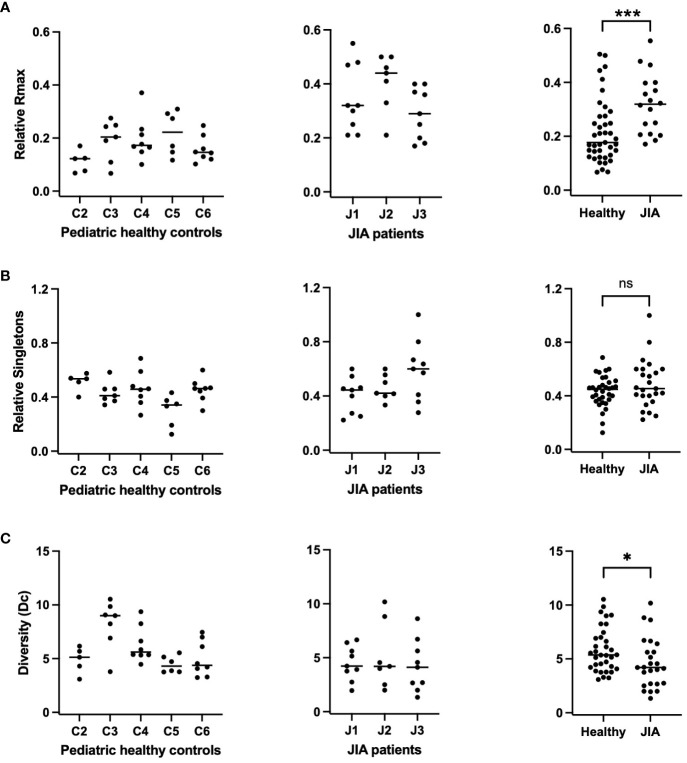
Relative R_max,_ relative singletons, and diversity of the RS/RA TCR βV19 repertoire in JIA patients compared to pediatric healthy controls. RS clonotypes analyzed multiple times over a 3-year period for each subject. Each datapoint represents data collected at 1 timepoint. **(A)** Relative R_max_ (R_max_/M) for pediatric healthy controls (panel 1), JIA patients (panel 2), and summary of data (panel 3). **(B)** Relative singletons (Ns/N) for pediatric healthy controls (panel 1), JIA patients (panel 2) and summary of data (panel 3). **(C)** Diversity for pediatric healthy controls (panel 1), JIA patients (panel 2) and summary of data (panel 3). Comparisons of pediatric healthy controls vs JIA patients was performed using Generalized Estimating Equation with repeated measures. * = *p<* 0.05; *** = *p<* 0.001; ns = not significant. Horizontal line indicates median of dataset.

We next plotted the same repertoire characteristics for all subject at each time point (**Figure 4**). In order to correct for individual sample size, R_max_ is reported as Relative R_max,_ or R_max_ divided by the total number of sequences (M); $\frac{Rmax}{M}$ and Singletons are reported as Relative Singletons, or Ns divided by the total number of clonotypes (N); $\frac{NS}{N}$ (27). The Relative R_max_ of each sample from each subject in the pediatric healthy controls and JIA cohort is shown in **Figure 4A**. The comparison of the Relative R_max_ between the two cohorts (**Figure 4A**, panel 3) shows that the JIA cohort has a higher Relative R_max_ than the pediatric healthy controls (0.17 vs. 0.33, *p<* 0.001). The Relative Singletons for the healthy and JIA cohorts are shown in **Figure 4B** and the comparison (**Figure 4B**, panel 3) shows that there is no significant difference in the healthy control and JIA cohorts. In summary, when analyzed over time, the Relative R_max_ was higher in the JIA RS/RA clonotype recall response than in the healthy controls, indicating that JIA patients had greater TCR repertoire unevenness.

### Repertoire Diversity is lower in JIA RS/RA clonotypes when measured over time

Clonotype Diversity (Dc) is a characteristic that incorporates both richness (N) and unevenness (R_max_/M) and can be estimated by $\frac{Rmax(N)}{\text{M}-1}$ . With the assumption that a system is robust and resilient to distortions if it is rich in species (clonotypes) and their abundance is uneven, deviations in R_max_ or N may be compensated by one another (31). Thus far we have shown that the JIA cohort displays decreased repertoire richness but greater repertoire unevenness when compared to pediatric healthy controls. In order to assess the extent of compensation, we calculated repertoire Diversity at all time points in controls and JIA patients (**Figure 4C**). We found that repertoire Diversity of JIA patients was lower than the pediatric healthy controls (p=0.03), (**Figure 4C**, panel 3). The lower Diversity found in the JIA patients is due to decreased antigen specific proliferative response of the M1_58–66_ RS/RA repertoire, as measured with an *in vitro* recall assay.

### The pattern of clonal unevenness fluctuates within JIA patients when measured over time

**Figure 5:**
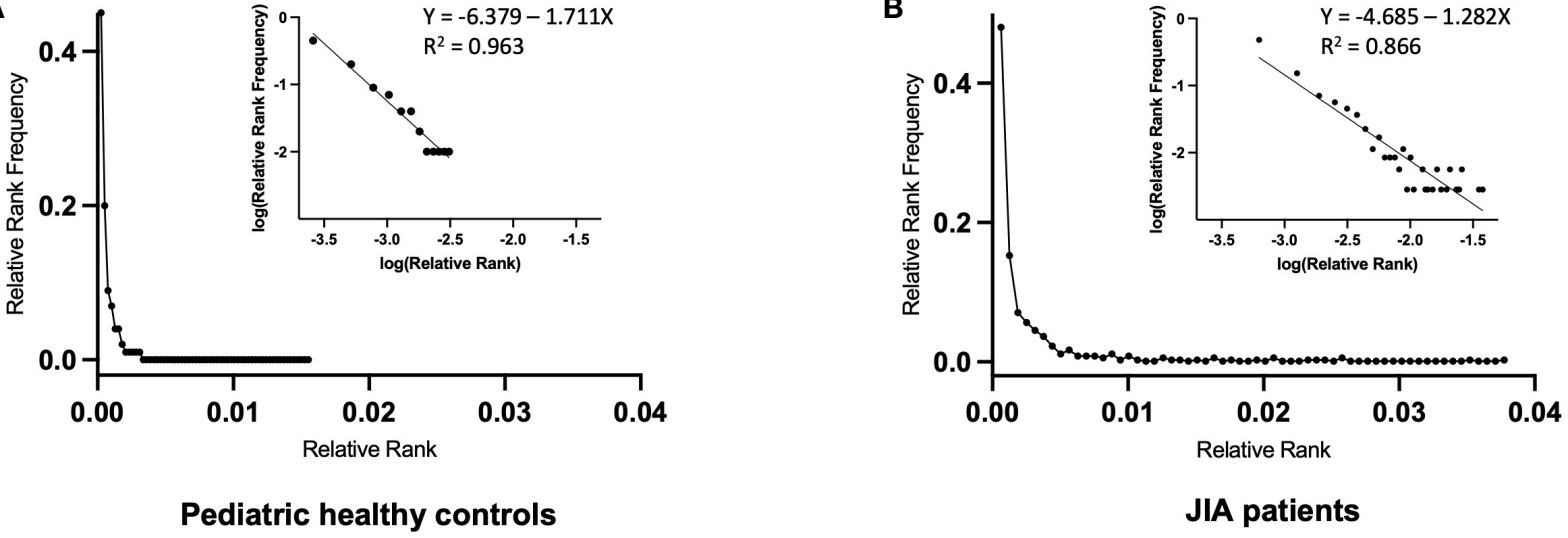
Relative Rank vs. Relative Rank Frequency of the RS/RA TCR βV19 repertoires of pediatric healthy controls and JIA patients. Repertoire distribution of RS/RA clones using the Rank Frequency approach. **(A)** Composite repertoire data from 5 pediatric healthy controls (C2, C3, C4, C5, C6) **(B)** Composite repertoire data from 3 JIA patients (J1, J2, J3). The inset graphs in A and B are the log/log transformation of the data with the superimposed trend line showing the best fit (R^2^ value).

Another way to visualize clonal unevenness is by plotting the distribution of all Ranks within the CDR3(11) RS/RA clonotype response. This is done using a modified Rank Frequency approach (30) in which Rank is plotted against Rank Frequency. To best depict differences in repertoires, these values are normalized by dividing by the number of sequences or clonotypes, respectively, present in the repertoire. Rank is normalized by dividing by the total number of sequences (M); $\frac{Rank}{M}$ , and reported as Relative Rank. Rank Frequency, or the number of times each rank occurs, is normalized by dividing by the total number of clonotypes (N); $\frac{Rank\;Frequency}{N}$ , and is reported as Relative Rank Frequency. The repertoire distribution can be visualized when Relative Rank Frequency is plotted on the *Y-axis* and the Relative Rank is plotted on the *X-axis*. **Figure 5** shows the combined data of repertoire distribution of clones from the 5 control subjects (C2, C3, C4, C5, C6) and the 3 JIA patients (J1, J2, J3) with RS/RA clonotypes at all study time points (**Figures 5A, B**, respectively). For both populations, the graph shows the clones with the lowest Relative Rank have the highest frequency. Indeed, there is little difference in the number of Singleton clonotypes between the JIA and healthy control cohort. A similar result has been reported for healthy adult controls (19, 27, 30). However, the JIA cohort repertoire is characterized by higher Ranks, as indicated by the longer tail along the x-axis (**Figure 5B**) and consistent with the higher Relative R_max_ in the JIA cohort.

The graphs of Relative Rank Frequency and Relative Rank are compatible with a single component power law-like distribution and plotting a log/log transformation of the data (log y = log _a-b_ log x) yields a straight line (30). A normally unselected TCR repertoire has many neutral receptors and a small number of receptors with high affinity for a particular ligand. As the biological response to TCR engagement is cell division within the lymphocyte network, the resultant repertoire yields a power law-like distribution that is driven by affinity based selective cell division, with clonotype Rank describing the outcome. The superimposed trend line of the combined data shows a R^2^ value of 0.96 for the 5 control subjects (C2, C3, C4, C5, C6) (**Figure 5A**, inset) and a R^2^ value of 0.86 for the 3 JIA patients (J1, J2, J3) (**Figure 5B**, inset). The slopes of the log/log transformation for the 3 JIA patients and 5 healthy controls are significantly different (p<0.0001). The shallower slope in the JIA cohort is driven by a lower number of expanded clones at each Rank Frequency, with a few unevenly distributed clones achieving a high level of expansion. In other words, the clonal expansion following ex vivo restimulation in the JIA cohort uncovered a less diverse repertoire due to the decreased richness of the response.

### Unique RS/RA clonotype stability is maintained in JIA patients, however there is wide variation in the distribution of clonotype expansion over time

**Figure 6:**
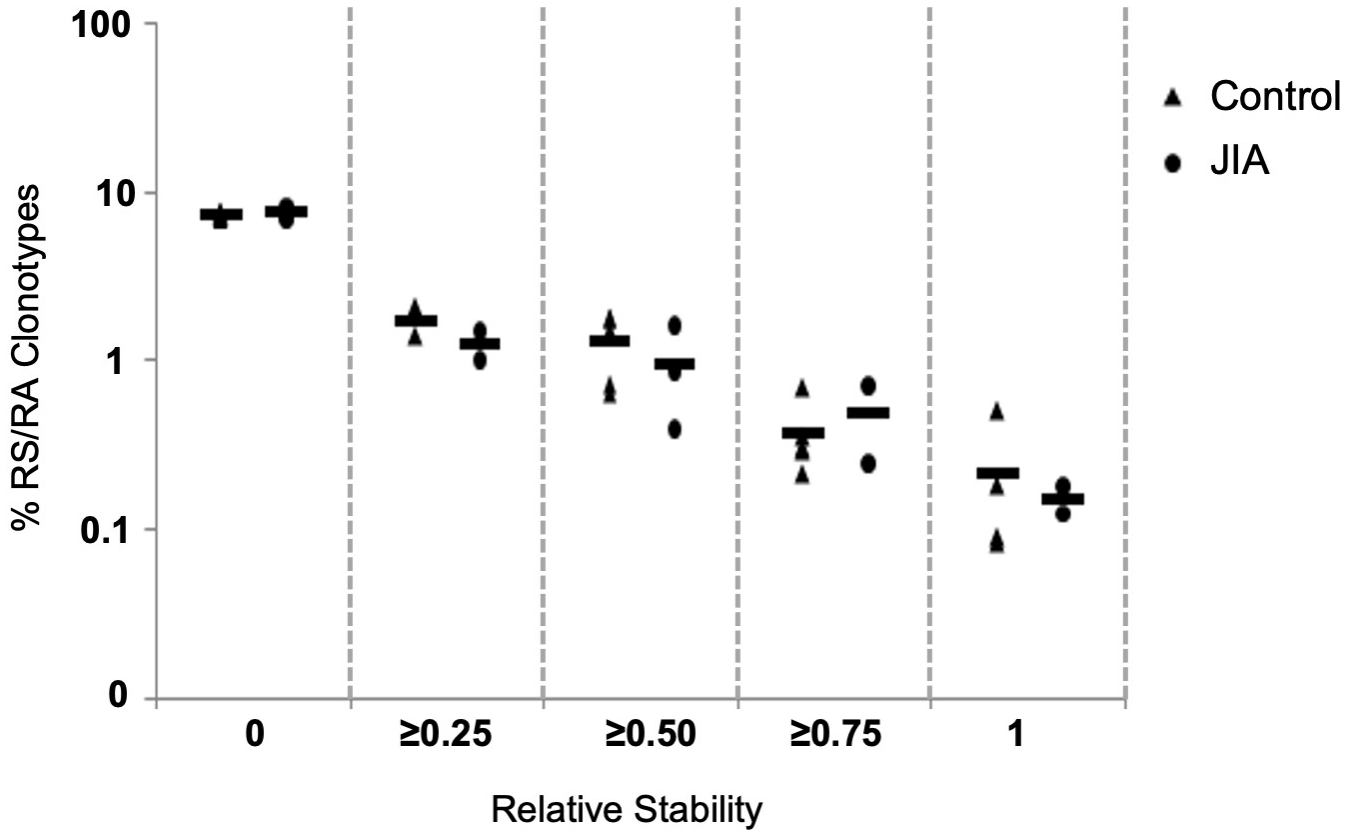
Clonotype stability in pediatric healthy controls and JIA patients. Clonotype temporal overlap. The percent of RS/RA encoding clonotype overlap between measurements taken at different time points. Since the number of time points varied between individuals, the percent of overlap was normalized based on number of each individual’s time points. The *X-axis* categorizes the percent of overlap, and the *Y-axis* shows the percent of clonotypes in each category. Control n = 6, JIA n = 5.

In order to assess clonotype temporal stability within the repertoire, we calculated the temporal overlap of clonotypes within the healthy control and JIA repertoires. The stability of a clonotype is defined as the number of times it was observed across repeated measurements. Clonotypes observed at all times analyzed are considered stable and clonotypes observed only once (with no temporal overlap) are considered unstable. Thus, clonotype temporal overlap is defined as the number of time points at which a clonotype was observed minus one; (number of times observed−1) (30), as a clonotype observed only once has no temporal stability. As the measurement of temporal stability is based on multiple time points, more time points should provide a better estimation of stability. To compensate for small differences in the number of time points, we report the Relative Stability of a clonotype, which is calculated stability; $\frac{\left(\text{number of times observed }–\;1\right)}{\left(\text{maximum possible number of times observed }–\;1\right)}\;$ . Using Relative Stability, an observation at one time is equal to a stability of 0 and an observation at all times is equal to a Relative Stability of 1. The Relative Stability was divided into 5 categories: (0), (≥ 0.25), (≥ 0.50), (≥ 0.75), (1). Only about 1% of all clonotypes were found in half of all available measurements (Relative Stability of ≥ 0.5). Importantly, the stability of responding clonotypes was similar in both healthy pediatric controls and JIA patients (**Figure 6**).

**Figure 7:**
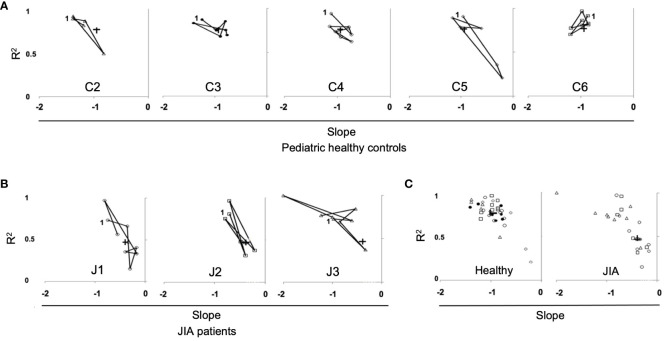
Repertoire distribution of RS/RA clones over time in pediatric healthy controls and JIA patients. Repertoire distribution of RS/RA clones over time showing a plot of slope versus R^2^ for all time points. **(A)** Pediatric healthy controls, **(B)** JIA patients, **(C)** Combined data from pediatric healthy control subjects (panel 1), (C2 = triangle, C3 = black circle C4 = white circle, C5 = diamond, C6 = square) and combined data from JIA patients (panel 2), (patient J1 = circle, J2 = square, J3 = triangle). + = the average value and “1” indicates the first time point.

The data in **Figure 6** measures the stability of the repertoire (recovery of unique clonotypes overtime). The stability of the immune response (reproducible distribution of clonal expansion when challenged) is another important measurement of repertoire dynamics. To measure the stability of a repertoire’s distribution of expanded clonotypes overtime, we plotted the slopes and R^2^ values of the log/log transformation of Relative Rank vs Relative Rank Frequency for each time point in each healthy control (**Figure 7A**) and each JIA patient (**Figure 7B**). For three of the five control subjects, time point data are well clustered and close to the cohort average, consistent with the idea that clonal expansion is reproducible with repeated antigenic exposure. In the other two healthy subjects (C2 and C5) there is some temporal variation in the repertoire shape. All three JIA subjects showed large changes in distribution of clonotypes in the repertoire over time. In aggregate, the slope vs R^2^ of the healthy control cohort is clustered around the mean (-0.95, 0.77) whereas there is less clustering around the mean (-0.62, 0.61) for the combined JIA cohort data (**Figure 7C**). The data illustrate greater fluctuations in the repertoire shape of the JIA cohort.

### In JIA patients the percent of IFNγ producing CD8^+^ βV19^+^clonotypes in response to M1_58–66_ is variable between visits and decreased compared to the healthy controls

**Figure 8:**
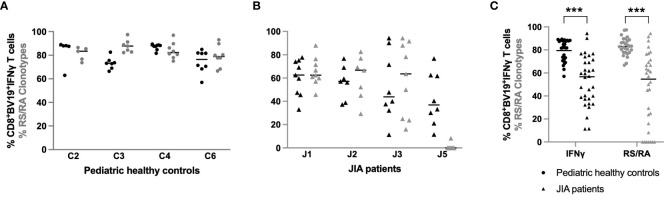
Percent of IFN-*γ* producing CD8^+^βV19^+^ T cells in pediatric healthy controls and JIA patients. Percent of CD8^+^βV19^+^IFN*γ* producing cells (CD8^+^βV19^+^IFN*γ* ^+^/total CD8^+^βV19^+^ T cells) in response to M1_58–66_ peptide obtained in **(A)** four pediatric healthy controls and **(B)** 4 JIA patients with sample available. **(C)** Combined data of percent IFN*γ*-producing CD8^+^βV19^+^cells and percent RS/RA clonotypes from healthy controls (circles) and JIA patients (triangles). Comparisons of pediatric healthy controls vs JIA patients was performed using Generalized Estimating Equation with repeated measures. *** = *p<* 0.001. Horizontal line indicates median of dataset.

The data thus far has shown different characteristics in CDR3 length and clonotype diversity in the CD8^+^ T cells expansion response to M1_58–66_ between JIA patients and healthy pediatric controls. Whether CD8^+^ T cells clones from JIA patients are functional against M1_58–66_ is a critical parameter to explore in order to understand adaptive cell-mediated immunity to this antigen. To address this question, IFNγ production was measured in response to ex-vivo stimulation of CD8^+^ T cell clones with the M1_58–66_ influenza peptide. We plotted the percentage of IFNγ producing CD8^+^βV19^+^ interferon producing cells for healthy controls (C2, C3, C4, C6) and JIA patients (J1, J2, J3, J5), as these patients had adequate sample available, at each study time point (**Figures 8A, B**, respectively). The percent of RS/RA clonotypes (**Figure 2**) is also replotted for purposes of comparison. The control cohort data is characterized by tight clustering of both the IFNγ production and the percent of RS/RA-encoding clonotypes above 60%. Conversely, the JIA data show wide intra-subject variability with extensive spreading of both the percent of RS/RA clonotypes and the percent of CD8^+^Vβ19^+^ IFNγ producing cells. When plotting the aggregate data from Figure A and B, JIA patients had a lower percent of IFNγ producing clonotypes as compared to healthy controls (p<0.001) (**Figure 8C**, panel 1). Of note, JIA samples had no difference in the absolute number of circulating CD8^+^ T cells and the frequency of CD8^+^ T cells was stable over time (data not shown).

## Discussion

The ability to mount an adequate immune response in patients with aberrant immunity or on immunosuppressive medications has been examined in multiple studies. These studies have largely been focused on the generation of antibody responses in adults and have driven recommendations for vaccination in these adult populations. In contrast, few studies have focused on antigen-specific memory T cell responses in children (38). Here we sought to assess the T cell recall response to the influenza antigen, M1_58–66_, in JIA patients receiving immunosuppressive therapy compared to pediatric healthy controls. We found the T cell repertoires of JIA patients exhibit several significantly different characteristics from healthy pediatric controls, including greater clonal unevenness, wider variability in repertoire distribution over time, a reduction in IFNγ-producing RS/RA clonotypes, and an overall lower level of clonal diversity owing to reduced clonal richness. When considering the T cell repertoire attributes at the clonal level, our study suggests that an antigen specific T cell memory response can be compromised in children with JIA on immunosuppressive medications. These data support the necessity of a larger study, one that targets not only influenza, but other infectious agents which utilize vaccination for protection.

The recall response of children with JIA was characterized by reduced clonal richness. In the JIA patients, the frequency of CDR3(11) clones was significantly less than the pediatric control cohort, the distribution of CDR3 length in the JIA patients was more similar to that of naïve T cells, and JIA patients had lower M1_58–66_ specific RS/RA clonotype production. Although the two JIA patients (J4 and J5) who produced almost no RS/RA clonotypes had high disease activity, uncontrolled inflammation and immune dysregulation cannot fully account for the lack of clonal richness. This is especially evident as patient J1 also had high disease activity but produced an RS/RA clonotype response similar to healthy controls. The intra-subject variability of RS/RA clonotype response over time is also visually evident when compared to the healthy controls. It is notable that all 5 JIA patients were receiving methotrexate, a dihydrofolate reductase inhibitor which increases T cell sensitivity to apoptosis. We speculate that iatrogenic immunosuppression may be the culprit of decreased clonal richness in JIA patients, while the wide variation in the percentage of RS/RA clonotypes within patients could be related to the periodic timing of immunosuppressive medication.

JIA patients also had a higher Relative R_max_ indicating that the clonal unevenness and distribution of RS/RA clonotypes in JIA patients is greater than that seen in healthy individuals. It is plausible that the JIA patients have a more focused antigen response, with overall fewer clones that occur at higher frequencies. For instance, JIA patients may have a few high affinity clones that are more resilient, while clonotypes of intermediate affinity may not expand as robustly in an immunosuppressed state, and thus drop to lower Ranks. This hypothesis may in part explain the flattening of the slope in the log/log transformation of the Relative Rank and Relative Rank Frequency seen in the JIA cohort; clones with intermediate affinity do not expand past intermediate Ranks and those with high affinity are highly expanded. Indeed, the direct relationship between a clonotype’s frequency, its avidity for the MHC—peptide, and its ability to perform effector functions has been described in other studies (39–41). The high frequency clonotypes may constitute functional clonotypes whereas lower frequency clonotypes represent those that can survive in culture or replicate at low levels but have not achieved stable memory effector status. Taken together, our data in the JIA cohort demonstrate that despite a greater degree of clonal unevenness (Relative R_max_), there is decreased clonal richness (N), ultimately resulting in a repertoire characterized by decreased diversity; $Dc=\frac{N*Rmax}{M-1}$ , when compared to healthy controls.

Interestingly, data from a longitudinal study of 3 HLA-A2 adults over a period of 7–10 years showed similar age-related changes in the M1_58–66_ specific T cell repertoire (31). With aging, healthy adults had a decline in the number of RS/RA-clonotype singletons with a shift of existing RS/RA clonotypes into higher frequencies (31). We previously hypothesized that this small amount of high ranking clonotypes may represent a percentage of T cells found in the circulation at all times, which is compatible with a stable circulating depot of cells. In this scenario, Rank would not only be a function of previous expansion, but also of accessibility in a mature memory repertoire (29). This theory was supported by our prior observation that the high Ranking clonotypes in healthy middle-aged adults were consistently and strongly associated with maximum stability. In contrast, this level of stability was not observed in JIA patients. Although we found clonotype stability in JIA patients to be similar to pediatric healthy controls, both cohorts display lower levels of stability than the adult repertoire (29), with none of the highest ranking clonotypes present at all times. This would suggest that in JIA, a high Relative R_max_ is not a function of accessibility nor a function of stability in the memory repertoire. Rather, this selective expansion is likely a proxy for the avidity of the TCR—peptide—MHC interaction influenced by chronic disease and/or immunosuppressive medication.

Analysis of the repertoire distribution of RS/RA clones over time also revealed greater temporal variation in the repertoire shape in JIA patients compared to the healthy control cohort. Both the slopes and R^2^ values lacked clustering around the average, often with lower R^2^ values in the JIA cohort. Thus, not only was the clonotype distribution more variable over time in the JIA patients, but also the repertoire shape at one time point was not as predictable as in the healthy controls. This indicates that clonal unevenness may not be maintained in JIA patients overtime, further emphasizing the decreased clonal diversity within this patient population. It is unclear if the variability in clonotype distribution represents a more directed (higher R_max_) RS/RA clonotype antigen response, a shift toward non-RS/RA clonotypes, or a combination of these factors in patients with JIA.

Functional analysis also showed that patients with JIA had significantly less interferon producing CD8^+^βV19^+^ cells following M1_58–66_ stimulation. Unlike the control cohort, some patients with JIA had less correlation between the percentage of RS/RA clonotypes and the percentage of IFNγ producing CD8^+^βV19^+^ cells at each visit. In fact, patient J5 displayed high IFNγ production despite a low frequency of RS/RA clones, suggesting recruitment of functional non-RS/RA M1_58–66_ specific clonotypes. This is particularly noteworthy as healthy adults have displayed recruitment of functional non-RS/RA clonotypes against M1_58–66_ with aging (29), again highlighting repertoire similarities between JIA patients and older individuals. Overall, the data suggests that IFNγ producing Vβ19 CD8^+^ T cells are decreased in JIA patients as compared to pediatric healthy controls.

There are several limitations to the current study. All patients received influenza immunization at baseline, 12, 24, and 36-month visits with various seasonal trivalent vaccines administered. Furthermore, there is no indication of how many times subjects may have been exposed to influenza prior to the study. In addition, clonotype expansion was assessed *in vitro*, in response to M1_58–66_ stimulation. This *in vitro* recall assay is unlikely to fully reflect the capacity for antigen-specific T cell responses to M1_58–66_ *in vivo*, as the two environments are functionally distinct. In theory, a reduced capacity to respond *in vitro* should apply equally to both patients and controls. However, it is possible that there is selective reduction in the *in vitro* proliferative potential of T cells from JIA patients. Thus, assessing clonotype expansion *in vitro*, in response to M1_58–66_ stimulation, may not fully reflect clonal diversity in response to vaccination *in vivo*. In other words, the lower diversity detected in our population may in part arise from differences between *in vivo* and *in vitro* expansion in JIA patients.

Another caveat is that our analysis assumes that all Vβ19^+^ CDR3(11) RS/RA clonotypes detected in our *in vitro* recall assay are derived from T cells that are specific to the M1_58–66_ peptide. Although this approach is supported by several studies (17, 18), we cannot exclude the possibility that non-specific T cells with a Vβ19^+^ CDR3(11) RS/RA sequence survive the culture conditions and contribute to measurements of diversity. As such cells would not expand in culture, their contribution to measurements of diversity are likely to be limited.

Furthermore, arthritis treatment was not standardized between patients. Although all patients received methotrexate, other immunosuppressive medications differed. Lastly, our patient and control cohorts were small; two JIA patients were omitted from the analysis of repertoire characteristics as they did not generate RS/RA clonotypes and our small sample size did not allow sub-analysis of disease factors, such cJADAS scores. Lastly, one healthy control (C1) who was 6-years-old at study start had very few RS/RA clonotypes, possibly due to polymorphism in an immune response gene, but more likely due to lack of an influenza exposure.

In summary, our data show that the ability to generate and maintain CD8^+^ T cell memory in patients with JIA on immunosuppressive therapy is significantly altered. Importantly, patients with JIA have less repertoire diversity owing to a reduction in clonal richness. The reduction in repertoire diversity occurs in spite of increased clonal unevenness. This molecular phenotype mimics that seen with aging but displays less stability than expected of a mature repertoire in an aged individual. It can be postulated that disease activity, treatment with immunosuppression, or a combination of the two may be driving the differential clonal expansion. These repertoire changes may have a functional association, as effector cytokine production (IFNγ) in JIA patients is reduced related to controls. Furthermore, the extent to which recruitment of functional non-RS/RA clonotypes contributes to the IFNγ response in JIA patients is unclear. Indeed, TCR diversity is associated with effective control of viral infections, although data regarding an increased infection risk in JIA is mixed (42–44). Notably, our data support existing literature detailing decreased immunogenicity of influenza vaccination due to methotrexate (45, 46).

Our work is particularly relevant in understanding the immune response to vaccination in immunocompromised individuals. Specifically, variability in the CD8^+^ T cell response may be an important consideration when providing vaccine recommendations and anticipatory guidance to patients with JIA on immunosuppressive therapy.

## Data availability statement

The original contributions presented in the study are included in the article/**Supplementary Material**, further inquiries can be directed to the corresponding author/s.

## Ethics statement

The studies involving humans were approved by Children’s Wisconsin Institutional Board Review. The studies were conducted in accordance with the local legislation and institutional requirements. Written informed consent for participation in this study was provided by the participants’ legal guardians/next of kin.

## Author contributions

SS: Conceptualization, Formal analysis, Writing – original draft, Writing – review & editing. DH: Data curation, Formal analysis, Methodology, Writing – original draft. JG: Conceptualization, Writing – review & editing. JV: Data curation, Writing – review & editing. JN: Data curation, Writing – review & editing. MY: Formal analysis, Methodology, Writing – review & editing. EN: Formal analysis, Investigation, Methodology, Writing – review & editing. EH: Writing – review & editing, Data curation. MD: Writing – review & editing, Formal analysis. KY: Writing – review & editing, Formal analysis. JG: Writing – review & editing, Conceptualization, Formal analysis, Project administration, Funding acquisition, Resources, Supervision. CW: Writing – review & editing, Conceptualization, Methodology, Data curation, Project administration, Funding acquisition, Resources, Supervision.

## Glossary

**Table:**

**JIA**	Juvenile Idiopathic Arthritis
**TCR**	T cell receptor
**CDR3**	Third complementarity determining regions
**R**	Arginine
**A**	Alanine
**S**	Serine
**M**	Total number of observed CDR3(11) TCR sequences
**N**	Total number of observed unique CDR3(11) TCR sequences (clonotypes), proxy for richness
**R_max_**	Maximum number that an individual clonotype appeared
**Ns**	Number of clonotypes that appeared only once (Singleton Clonotypes)
**PGA**	Physician global assessment
**JC**	Joint count
**cJDAS**	clinical Juvenile Arthritis Disease Activity Score
**IV**	intravenous
**D_c_**	Diversity
**Clonal Richness**	Total number clonotypes, (N)
**Clonal Unevenness**	The maximum Rank within a repertoire divided by the total number of sequences: $\frac{Rmax}{M}$
**Clonotype**	Unique nucleotide CDR3(11) TCR sequence
**Clonotype Stability**	Number of times a clonotypes is observed across repeated measurements
**Diversity**	An indicator of both clonal richness and clonal unevenness, $\frac{Rmax(N)}{\text{M}-1}$
**Rank**	Number of times a clonotype is observed within a TCR repertoire
**Rank Frequency**	Number of times a Rank is observed
**Relative Rank**	Rank divided by the total number of sequences: $\frac{Rank}{M}$
**Relative Rank Frequency**	Number of times a Rank is observed divided by the total number of clonotypes: $\frac{Rank\;Frequency}{N}$
**Relative Clonotype Stability**	$\frac{\text{number of times observed }–\;1}{\text{maximum possible number of times observed }–\;1}$
**Relative R_max_**	A proxy for unevenness, R_max_ divided by the total number of sequences: $\frac{Rmax}{M}$
**Relative Singletons**	Singletons (Ns) divided by the total number of clonotypes: $\frac{Ns}{N}$
