# Corrigendum: Patients with juvenile idiopathic arthritis have decreased clonal diversity in the CD8^+^ T cell repertoire response to influenza vaccination

**DOI:** 10.3389/fimmu.2024.1446946

**Published:** 2024-06-19

**Authors:** Sara E. Sabbagh, Dipica Haribhai, Jill A. Gershan, James Verbsky, James Nocton, Maryam Yassai, Elena N. Naumova, Erin Hammelev, Mahua Dasgupta, Ke Yan, Jack Gorski, Calvin B. Williams

**Affiliations:** ^1^ Division of Rheumatology, Department of Pediatrics, Medical College of Wisconsin, Milwaukee, WI, United States; ^2^ Divison of Hematology/Oncology, Department of Pediatrics, Medical College of Wisconsin, Milwaukee, WI, United States; ^3^ Versiti Wisconsin, Blood Research Institute, Milwaukee, WI, United States; ^4^ Division of the Nutrition Epidemiology and Data Science, Friedman School of Nutrition Science and Policy, Tufts University, Boston, MA, United States; ^5^ Division of Quantitative Health Sciences, Department of Pediatrics, Medical College of Wisconsin, Milwaukee, WI, United States

**Keywords:** CD8+ T cells, T cell repertoire, influenza vaccination, clonotypes, juvenile idiopathic arthritis, clonotype diversity

In the published article, there was an error in the **Funding** statement. The funding statement for the D.B. and Marjorie Reinhart Family Foundation was misspelt as “D.B. and Majorie Reinhart Family Foundation”. The correct **Funding** statement appears below.

**FUNDING**

The author(s) declare that financial support was received for the research, authorship, and/or publication of this article. This study was supported by R01-AI26085 and NO1-AI50032 NIAID NIH HHS/United States. D.B. and Marjorie Reinhart Family Foundation. Children’s Wisconsin.

The authors apologize for this error and state that this does not change the scientific conclusions of the article in any way. The original article has been updated.

