# Supplementary material for: Patients with juvenile idiopathic arthritis have decreased clonal diversity in the CD8+ T cell repertoire response to influenza vaccination
Source: Front Immunol. 2024 May 30;15:1306490. doi: 10.3389/fimmu.2024.1306490 (PMC11169902; doi:10.3389/fimmu.2024.1306490)
Supplement: Supplementary file 1 [file DataSheet_1.pdf]

Supplemental Table 1: Trivalent influenza vaccines administered to JIA patients and healthy controls											
**Season; *strains***	**JIA patients**					**Healthy controls**					
2006-2007<br>*A/New Caledonia/20/99*<br>*A/Wisconsin/67/2005*<br>*B/Malaysia/2506/2004*	J1		J3		J5						
2007-2008<br>*A/Solomon Islands/3/2006*<br>*A/Wisconsin/67/2005*<br>*B/Malaysia/2506/2004*	J1*	J2	J3	J4	J5	C1	C2	C3	C4	C5	C6
2008-2009<br>*A/Brisbane/59/2007*<br>*A/Brisbane/10/2007*	J1	J2	J3	J4	J5	C1	C2	C3	C4	C5	C6
2009-2010<br>*A/Brisbane/59/2007*<br>*A/Brisbane/10/2007*<br>*B/Brisbane/60/2008*	J1	J2	J3	J4	J5	C1*	C2	C3	C4	C5	C6*
2010-2011<br>*A/California/7/2009*<br>*A/Perth/16/2009*<br>*B/Brisbane/60/2008*	J1	J2		J4		C1	C2	C3	C4	C5	C6
Study participants received the annual trivalent non-adjuvanted inactivated vaccine (Fluzone, Sanofi-Pasteur MSD, Madrid, Spain) for at least 4 consecutive years. *self-reported.											
